# Cardiovascular disease risk factors and markers of oxidative stress and DNA damage in leprosy patients in Southern Nigeria

**DOI:** 10.1371/journal.pntd.0008749

**Published:** 2020-10-12

**Authors:** Iya Eze Bassey, Inyeneobong Ernest Inyang, Uwem Okon Akpan, Idongesit Kokoabasi Paul Isong, Bassey Edward Icha, Victoria Micheal Ayawan, Racheal Ekanem Peter, Hopefaith Adode Itita, Prince Ukam Odumusor, Eyoanwan Graziani Ekanem, Okon Ekwerre Essien

**Affiliations:** 1 Department of Medical Laboratory Science, Faculty of Allied Medical Sciences, College of Medical Sciences, University of Calabar, Calabar, Cross River State, Nigeria; 2 Department of Chemical Pathology, University of Calabar Teaching Hospital, Calabar, Cross River State, Nigeria; 3 Department of Microbiology, Faculty of Natural Science, Caritas University, Amorji Nike, Enugu State, Nigeria; 4 Department of Public Health, Faculty of Allied Medical Sciences, College of Medical Sciences, University of Calabar, Calabar, Cross River State, Nigeria; 5 Department of Microbiology, University of Calabar Teaching Hospital, Calabar, Cross River State, Nigeria; 6 Department of Internal Medicine, Faculty of Medicine, University of Calabar, Calabar, Cross River State, Nigeria; Federal University of Ceará, Fortaleza, Brazil, BRAZIL

## Abstract

Leprosy reduces quality of life of affected persons. Oxidative stress caused by reactive oxygen species may play a vital role in the pathogenesis of leprosy. This study evaluated anthropometric indices, fasting plasma glucose (FPG), lipid profile, total antioxidant capacity (TAC), total plasma peroxide (TPP), oxidative stress index (OSI), malondialdehyde (MDA), glutathione (GSH) and 8-hydroxy-2-deoxyguanosine (8-OHdg) in leprosy patients. Sixty test participants of both genders, aged 18–65years and diagnosed of multibacillary leprosy and 30 apparently healthy controls were consecutively recruited for this study. The test participants comprised of 30 patients on multidrug therapy (MDT) and 30 patients relieved from therapy (RFT). Body mass index (BMI), Waist-hip ratio (WHR), FPG, lipid profile, TAC, TPP, OSI, MDA, GSH and 8-OHdg were determined using appropriate methods. Data were analyzed using Analysis of variance; p<0.05 was considered statistically significant. The MDT group had significantly lower BMI (p = 0.0001), Total cholesterol (p = 0.001), HDL-C (p = 0.019), LDL-C (p = 0.005), TAC (p = 0.0001) and higher TPP (p = 0.001), MDA (p = 0.0001), OSI (p = 0.005) and 8-OHdg (p = 0.035) compared to the controls. The RFT group had significantly lower BMI (p = 0.001) Total cholesterol (0.0001), HDL-C (p = 0.006) LDL-C (p = 0.0001), TAC (p = 0.001) and higher WHR (p = 0.010), VLDL-C (p = 0.035), TG (p = 0.023) Atherogenic index of plasma (p = 0.0001) and TPP (p = 0.001), MDA (p = 0.0001) compared to the control group. GSH levels correlated negatively with duration of treatment (r = -0.401, p = 0.028). This study has shown that there is oxidative stress in multibacillary leprosy patients irrespective of drug treatment status. This study also shows that leprosy patients relieved from treatment may be susceptible to cardiovascular events. Antioxidants supplementation may be beneficial in the treatment of leprosy and clinical follow up on patients relieved from treatment may also be necessary to monitor health status and prevent development of cardiovascular events.

## Author summary

This study shows that there are lower levels of total antioxidant capacity and higher levels of total plasma peroxide, malondialdehyde in leprosy patients undergoing multidrug therapy and those relieved from treatment and higher levels of 8-OHdg and oxidative stress index in leprosy patients undergoing multidrug therapy. This is suggestive of increased oxidative stress, in multibacillary leprosy patients irrespective of drug treatment status and increased oxidative DNA damage in those undergoing multidrug therapy. Antioxidants supplementation may be beneficial in the treatment of leprosy to protect against the effects of oxidative stress and DNA damage. Leprosy patients relieved from treatment may be susceptible to cardiovascular events as shown by higher levels of VLDL-cholesterol, triglycerides and atherogenic index of plasma observed in that group compared to controls. It therefore points to the need to monitor cardiovascular comorbidities in patients on multidrug therapy and those released from therapy.

## Introduction

Leprosy or Hansen’s disease is a chronic debilitating disease caused by *Mycobacterium leprae* that affects the skin, peripheral nerves, mucosa of the upper respiratory tract, and the eyes causing progressive and permanent disabilities if not treated [1]. Although Leprosy was eliminated as a public health problem (i.e. a disease burden of < 1 case per 10 000 persons) globally in the year 2000, leprosy still remains a global public health issue [2]. Each year about 200–300 thousand people are diagnosed of leprosy and about 2–3 million people are disabled because of it [3]. Official figures from 150 countries from 6 WHO regions show that globally 7, 607, 837 persons are infected with leprosy [4]. A global registered prevalence of 192, 713 cases was reported in 2017, an increase by 20, 765 cases over that in 2016, and 210, 671 new cases were detected in the same year [5].

In Nigeria a registered prevalence of 3,234 cases was reported in 2015 and 2,892 new cases were detected in the same year [6]. With this, Nigeria ranked third among African countries with the highest burden of the disease [7]. However in 2017, prevalence of 11, 230 cases was reported and 2,447 new cases detected, with a total of 195, 875 persons infected with leprosy in the country; making Nigeria first among African countries with the highest burden of the disease in 2017 [5]. Nigeria Center for Disease Control (NCDC) moreover maintains that over 3,500 people are diagnosed with leprosy yearly in the country with about 25% of victims having some degree of disability [8].

The pathogenesis of this neurodegenerative disease is not fully understood [9]. These leprosy-associated disabilities potentially caused by damage to the peripheral nerves have been linked to a number factors including oxidative stress [10]. Excessive production of reactive oxygen species (ROS) is the major precursor of Oxidative Stress (OS). Oxidative Stress is a condition associated with an increased rate of metabolic impairment and cellular damage occurring due to derangement in the balance between ROS and the body’s antioxidant system [11]. It is suggested that the possible reason for decreased antioxidant status in leprosy cases may be increased production of ROS via cell mediated immunity, as well as the free radical producing ability of drugs used in multidrug therapy (MDT) of leprosy [12]. Of the various mechanisms that influence the pathogenesis of leprosy, oxidative stress may well be important; however, only a few studies have been done to assess the status of oxidative stress in leprosy patients via markers of oxidative stress such as Glutathione (GSH), Superoxide dismutase (SOD), and lipid peroxidase product, Malondialdehyde (MDA). 8-hydroxy-2deoxy guanosine (8-OHdG) is a DNA nucleoside which is an oxidative derivative of guanosine [10]. Measurement of the levels of 8-OHdG is important as a biomarker of oxidative stress causing cellular DNA damage. Increased levels of 8-OHdG is associated with a lot of pathologic conditions like cancer, diabetes, and hypertension [13].

Cardiovascular diseases (CVD) are the leading cause of death and disability worldwide. Together they resulted in 17.9 million deaths in 2015 up from 12.3 million in 1990 and represented 31% of all global deaths in 2016 [4]. Growing evidence indicates that chronic and acute over production of reactive oxygen species under pathophysiologic conditions is integral in the development of cardiovascular diseases [14]. Majority of cardiovascular disease results from complications of atherosclerosis, and an important initiating event for atherosclerosis may well be the oxidation of low-density lipoprotein (LDL) and the transport of oxidized low-density lipoprotein (Ox-LDL) across the endothelium into the artery wall [15]. Dyslipidaemia, hyperglycaemia, and insufficient physical activity are cardiovascular risk factors that have been associated with leprosy [16, 17].

In spite of the global interest in CVD and the growing interest in oxidative stress and its role in the aetiology of many diseases, very few studies on these have been conducted among people affected by leprosy. The few studies evaluating oxidative status in leprosy patients were conducted in India and Brazil [11, 12, 16, 18] and none amongst African people affected with leprosy. There is also dearth of information regarding the link between leprosy and the risk of cardiovascular disease in Africans, hence the need for this study. The purpose of this study was to assess the cardiovasculardisease risk factors and markers of oxidative stress and DNA damage in a native black population of leprosy patients to see if there are any deleterious changes on these markers especially in those undergoing multiple drug therapy and those relieved from treatment. The study assessed anthropometric indices, fasting plasma glucose, lipid profile, total antioxidant capacity (TAC), total plasma peroxide (TPP), oxidative stress index (OSI), malondialdehyde (MDA), glutathione (GSH) and 8-hydroxy-2-deoxyguanosine (8-OHdg) in patients with leprosy.

## Methodology

### Ethics statement

This study was carried out in accordance with the World Medical Association’s Declaration of Helsinki [19]. Ethical clearance was obtained from Health Research and Ethics Committee of the Akwa Ibom State Ministry of Health, Nigeria (Ref No.: MH/PRS/99/vol.IV/452). The study participants were informed of the nature of the research and written informed consent obtained as approved by the ethics committee before they were enrolled for the study.

### Study design and subject selection

A case-control study design was used for the study and was carried out in Ekpene Ebom, Etinan LGA, in Akwa Ibom State Nigeria. A total of 90 (male and female) participants were consecutively recruited for the study. They consisted of 60 known multibacillary leprosy patients as test subjects and 30 apparently healthy individuals who did not have leprosy as controls. They were aged between 18 to 65 years and were all Nigerians. The leprosy patients were recruited from outpatients and inpatients of the Leprosy Referral Hospital in Ekpene Ebom, and 30 apparently healthy individuals were recruited as control participants from Akwa Ibom State. The 60 test participants comprised of 30 patients on multidrug therapy (MDT) and 30 patients who have been relieved from treatment (RFT). Treatment modality for the patients was the administration of Dapsone 100 mg /daily, Rifampicin 600 mg/daily and Clofazimine 50 mg /daily for 24 months. The study was carried out from 1 September 2018 to 1 August 2019. A standard questionnaire was administered and sociodemographic data obtained.

### Measurement of anthropometric indices and blood pressure and definition of cut-off

Each participant’s weight (in kilograms) and height (in metres) were measured. A weighing balance was utilized to measure weight in kilograms and a stadiometer was used to measure height in meters. Body mass index (BMI) was calculated by as the ratio of the weight to the square of height (kg/m^2^). Normal range for BMI is 18–25 kg/m^2^. Obesity was defined as BMI ≥30 kg/m^2^. Waist circumference (in centimeters) was obtained by taking two measurements, one after inhalation and the other after exhalation at the midpoint between the top of the iliac crest and the bottom of the rib cage using an inelastic calibrated tape. Hip circumference measured at the hips and buttocks. Waist-to-hip ratio was defined as waist girth/hip circumference. Obesity was defined as Waist/hip ratio >0.9 (men), >0.85 (women). The blood pressure was measured on the right arm with a mercury sphygmomanometer (cuff size 12.5 X 40 cm) with the patient in a seated position and after a 5-minute rest. The systolic and diastolic blood pressures were recorded. Hypertension was defined as blood pressure ≥140/90 mmHg or the use of antihypertensive medications [20].

### Inclusion and exclusion criteria

The test participants were recruited not withstanding impairment/disability status. Leprosy patients who had just been diagnosed and had not started any treatment regimen, those refused their consent to participate and non-infected persons with any sign or symptoms of illness at the time of the study were excluded.

### Collection of sample

Seven millilitres (7ml) whole blood was collected aseptically by venepunture using a Terumo vacutainer (Terumo, Japan) from each participant after an overnight fast. Two millilitres (2ml) was dispensed into fluoride oxalate bottles for fasting plasma glucose estimation while five millilitres (5ml) was dispensed into a plain bottle, allowed to clot at room temperature, centrifuged at 3000rpm for 5minutes and serum extracted and and stored at -20°C.

### Estimation of analytes

#### Fasting plasma glucose (FPG) and lipid profile

Fasting plasma glucose (FPG) was estimated by the glucose oxidase method of Trinder, [21]. Total cholesterol (TC) was estimated by the method of Allain *et al*., [22] and Triglycerides by the method of Bucolo and David, [23]. Estimation of high density lipoprotein cholesterol (HDL-C) was by Precipitation and cholesterol determination method of Demacker *et al*., [24] and Allain *et al*., [22] respectively. All the kits were obtained from Randox (Antrim, United Kingdom). Low Density Lipoprotein Cholesterol (LDL-C) was calculated using Friedewald’s equation: LDL-C = TC—HDL-C—VLDL-C [25] while Very Low Density Lipoprotein Cholesterol (VLDL-C) was calculated using the equation: VLDL-C (mmol) = TG/2.2 provided TG was ≤ 4.5mmol/l [26]. Normal ranges for the variables were as follows: FPG— 3.5—5.5mmol/L; TC: 3.0—6.5mmol/L; HDL-C: 0.9—1.5mmol/L; LDL-C: 2.5—3.8mmol/L; VLDL-C: 0.4—0.6mmol/L; TG: 1.2mmol/L. Atherogenic Index Plasma was calculated according to the formula, log (TG/HDL-C) [27]. Subjects with AIP value of > 0.11 were considered at elevated cardiovascular risk.

#### Oxidative stress markers

Determination of glutathione (GSH) was based on the methodology described by Ellman, [28] and malondialdehyde (MDA) was by the method of Buege and Aust [29]. Total antioxidant capacity was determined by the method of Koracevic *et al*., [30]. Total plasma peroxide (TPP) was determined using the reaction of ferrous-butylated hydroxytoluene-xylenol orange complex ‘FOX2’ method of Miyazawa, [31] with minor modifications as described by Harma *et al*., [32]. The ratio of total plasma peroxide (TPP) to total antioxidant capacity (TAC) was calculated as the oxidative stress index, an indicator of the degree of oxidative stress.

$$\text{OSI}\;(\%)\;=\;[\text{TPP}(\mu \text{mol}/\text{l}\;{\text{H}}_{2}{\text{O}}_{2})\;\text{x}\;100)/\;[\text{TAC}\;\mu \text{mol}/\text{l}].$$

#### Marker of oxidative DNA damage

Estimation of 8-OHdG was done using Agisera’s ELISA kit no: AS152887 (Agrisera, Vännäs, Sweden). The Agrisera’s 8-OHdG ELISA is a competitive assay for the quantitative measurement of 8-OHdG. It utilizes an 8-OHdG coated plate and an HRP-conjugated antibody for detection which allows for an assay range of 0.94-60ng/L, with a sensitivity of 0.59ng/L. The 8-OHdG antibody used in this assay recognizes both the free 8-OHdG and the DNA incorporated 8-OHdG. All the analytes were estimated in the Chemical Pathology Laboratory of the University of Calabar Teaching Hospital, Calabar, Nigeria.

### Statistical analysis

Data was analyzed using the PAWstatistic 18, a statistical package from SPSS Inc, (Chicago IL, United States of America) and R version 4.0.0 from The R Foundation for Statistical Computing Platform, Vienna, Austria. Results were presented as mean ± standard deviation. Chi-squared test of independence was to test significant relationship between categorical variables. The assessment of the normality of the data was done using the Shapiro-Wilk’s test. Comparisons of groups were made using Students’ t-test and analysis of variance (ANOVA) and post hoc analysis using Least significant difference. Pearson’s correlation analysis was also done. The level of significance was set at 95% confidence interval, where p-value less than 0.05 (p<0.05) was considered as statistically significant. Graphs were created with Microsoft excel 2007 version (Microsoft Corporation, US).

## Results

Table 1 shows the sociodemographic data of leprosy patients on MDT, those RFT and controls. The groups have similar sex distribution, but those relieved from treatment are older than both the controls and those undergoing treatment. The occupation of the leprosy patients was mostly farming and petty trading. However, a large proportion (60%) of those relieved from treatment were unemployed. In the MDT group, a significantly large proportion (83.3%), compiled strictly to treatment regimen with only 16.7% (n = 5) lagging in compliance. There was no co-infection with HIV in the test group. The leprosy patients relived from treatment had a significantly higher (p = 0.0001) frequency of physical deformity as well as history of leprae reactions.

**Table 1:** Sociodemographic data of leprosy patients undergoing multidrug therapy, leprosy patients relieved from treatment and control.

Variable	Leprosy patients undergoing multidrug therapy n = 30	Leprosy patients relieved from treatment n = 30	Controls n = 30	P-value
**Age**	38.4± 16.80	49.8± 9.76^#^	38.0± 12.79	0.001
**Occupation**	Students 3 (10%) Traders 11(36.7%) Farmers 16 (53.3%)	Farmers 6(20%) Traders 6(20%) Unemployed 18(60%)	Students 7 (23.3%) Farmers 5(16.7%) Traders 5(16.7%) Civil servants 13(43.3%)	
**Gender**	Male 19 (63.3%) Female 11 (36.7%)	Male 22 (73.3%) Female 8 (26.7%)	Male 17 (56.7%) Female 13 (43.3%)	0.398
**Compliance with treatment**	Compliant: 25 (85%)* Not Complaint: 5 (15%)			
**Physical deformity**	4 (13.3%)	20(66.7%)	Nil	0.0001
**History of Leprae Reactions**	3(10%)	21(70%).	Nil	0.0001
**Duration of MDT (months)**	7.3±6.11 (Min (0.5); Max(23)			
**Duration relieved from treatment(yr)**		3.5±1.96 (Min (1.0); Max(8.0)		

Table 2 shows the comparison of anthropometric indices, blood pressures, lipid profile, atherogenic index of plasma and oxidative stress markers in leprosy patients undergoing multiple drug therapy, leprosy patients relieved from treatment and controls. The result shows that there was a significant variation in the levels of BMI (p = 0.0001), WHR (p = 0.033), DBP (p = 0.048), TC (p = 0.0001), HDL-C (p = 0.013), LDL-C (p = 0.001), VLDL-C (p = 0.047), TG (p = 0.037), AIP (p = 0.001), TAC (p = 0.0001), TPP (p = 0.001), OSI (p = 0.018), MDA (p = 0.0001), GSH (p = 0.036) and 8-OHdG (p = 0.015) among the groups. There was no significant variation (p>0.05) in the SBP and FPG levels among the groups. Due to the fact that the RFT were older than both controls and MDT group we adjusted for age by adding age as a covariate into the analysis and the results did not change.

**Table 2:** Anthropometric indices, blood pressures, lipid profile, atherogenic index of plasma and oxidative stress markers in leprosy patients undergoing multiple drug therapy (MDT), leprosy patients relieved from treatment (RFT) and controls.

Parameter	Leprosy Patients		Controls n = 30	p-value
	MDT n = 30	RFT n = 30		
BMI (kg/m^2^)	19.8 ± 4.30	20.3 ± 3.94	23.6± 2.58	0.0001*
Waist/Hip ratio	0.86 ±0.04	0.89 ±0.08	0.85 ±0.05	0.033*
Systolic BP (mmHg)	113.3 ±14.16	122.7 ±22.73	116.8 ±6.58	0.078
Diastolic BP (mmHg)	71.8 ±7.93	74.0 ±11.91	78.2 ±9.74	0.048*
FPG (mmol/L)	6.19 ± 7.25	4.38 ± 0.64	4.42 ± 0.47	0.173
Total cholesterol (mmol/L)	3.92 ± 0.86	3.46 ± 0.97	4.68 ± 0.71	0.0001*
HDL-cholesterol (mmol/L)	1.41 ± 0.39	1.37 ± 0.35	1.65 ± 0.41	0.013*
LDL-cholesterol (mmol/L)	2.05 ± 0.69	1.59 ± 0.92	2.61 ± 0.58	0.0001*
VLDL-cholesterol (mmol/L)	0.45 ± 0.25	0.56 ± 0.20	0.45 ± 0.14	0.047*
Triglycerides (mmol/L)	0.99 ± 0.54	1.24 ± 0.43	0.98 ± 0.30	0.037*
Atherogenic index of plasma	-0.18 ±0.20	-0.05 ±0.19	-0.23 ±0.18	0.001*
TAC (mmol)	1.20 ± 0.45	1.40 ± 0.50	1.94 ± 0.76	0.0001*
TPP (μmol/L)	111.8 ± 11.66	112.5 ± 8.97	104.2 ± 6.55	0.001*
Oxidative Stress index	12.0 ± 9.07	9.77 ±5.59	6.9 ±4.75	0.018*
MDA (mmol/L)	0.74 ± 0.18	0.87 ± 0.23	0.52 ± 0.10	0.0001*
GSH (mg/dL)	79.1 ± 20.34	79.2 ± 20.67	90.6 ± 29.70	0.111
8-OHdg (ng/L)	9.2 ± 7.98	5.67 ± 1.36	6.44 ± 1.80	0.015*

Results expressed as Mean ± SD

*significant at p<0.05

A post hoc analysis showed that the mean values of BMI (p = 0.0001), DBP (p = 0.016), TC (p = 0.001), HDL-C (p = 0.019) and LDL-C (p = 0.005) and TAC (p = 0.0001) were significantly lower while the mean values of TPP (p = 0.001), OSI (p = 0.005), MDA (p = 0.0001) and 8-OHdg (p = 0.035) were significantly higher in the MDT group when compared to controls. The mean values of BMI (p = 0.001), TC (p = 0.0001), HDL- C (p = 0.006), LDL-C (p = 0.0001) and TAC (p = 0.001) were significantly lower in the RFT group while the mean values of WHR (p = 0.010), VLDL-C (p = 0.035), TG (p = 0.023), AIP (p = 0.0001), TPP (p = 0.001) and MDA (p = 0.0001) were significantly higher in the RFT group when compared to the controls. Further comparison between the MDT and RFT group showed that TC(p = 0.041), LDL-C(p = 0.019) and 8-OHdg (0.006) levels were significantly lower while VLDL-C (p = 0.050), TG(p = 0.030), AIP (p = 0.011), MDA (p = 0.004) and were significantly higher in the RFT group when compared to the MDT group (Table 3).

**Table 3:** Comparison of anthropometric indices, blood pressures, lipid profile, atherogenic index of plasma and oxidative stress markers in leprosy patients undergoing multiple drug therapy (MDT), leprosy patients relieved from treatment (RFT) and controls using post hoc analysis.

Parameter	Groups		Mean Difference	Std Error	p-value
	MDT n = 30	Controls n = 30			
BMI (kg/m^2^)	19.8 ± 4.30	23.6± 2.58	**-3.800**	**0.951**	**0.0001**
Diastolic BP (mmHg)	71.8 ±7.93	78.2 ±9.74	-6.367	2.581	0.016
Total cholesterol (mmol/L)	3.92 ± 0.86	4.68 ± 0.71	-0.760	0.221	0.001
HDL-cholesterol (mmol/L)	1.41 ± 0.39	1.65 ± 0.41	-0.237	0.099	0.019
LDL-cholesterol (mmol/L)	2.05 ± 0.69	2.61 ± 0.58	-0.560	0.192	0.005
TAC (mmol)	1.20 ± 0.45	1.94 ± 0.76	0.733	0.151	0.0001
TPP (μmol/L)	111.8 ± 11.66	104.2 ± 6.55	7.643	2.401	0.001
Oxidative Stress index	12.0 ± 9.07	6.9 ±4.75	5.047	1.739	0.005
MDA (mmol/L)	0.74 ± 0.18	0.52 ± 0.10	0.210	0.046	0.0001
8-OHdg (ng/L)	9.2 ± 7.98	6.44 ± 1.80	2.772	1.290	0.035
	**RFT** **n = 30**	**Controls** **n = 30**			
BMI (kg/m^2^)	20.3 ± 3.94	23.6± 2.58	**-3.343**	**0.951**	0.001
Waist/Hip ratio	0.89 ±0.08	0.85 ±0.05	0.041	0.015	0.010
Total cholesterol (mmol/L)	3.46 ± 0.97	4.68 ± 0.71	-1.217	0.221	0.0001
HDL-cholesterol (mmol/L)	1.37 ± 0.35	1.65 ± 0.41	-0.277	0.099	0.006
LDL-cholesterol (mmol/L)	1.59 ± 0.92	2.61 ± 0.58	-1.020	0.192	0.0001
VLDL-cholesterol (mmol/L)	0.56 ± 0.20	0.45 ± 0.14	0.111	0.052	0.035
Triglycerides (mmol/L)	1.24 ± 0.43	0.98 ± 0.30	0.260	0.112	0.023
Atherogenic Index of plasma	-0.05 ±0.19	-0.23 ±0.18	0.182	0.049	0.0001
TAC (mmol)	1.40 ± 0.50	1.94 ± 0.76	-0.540	0.151	0.001
TPP (μmol/L)	112.5 ± 8.97	104.2 ± 6.55	8.330	2.401	0.001
MDA (mmol/L)	0.87 ± 0.23	0.52 ± 0.10	0.347	0.046	0.0001
	**MDT** **n = 30**	**RFT** **n = 30**			
Total cholesterol (mmol/L)	3.92 ± 0.86	3.46 ± 0.97	-0.457	0.221	0.041
LDL-cholesterol (mmol/L)	2.05 ± 0.69	1.59 ± 0.92	0.460	0.192	0.019
VLDL-cholesterol (mmol/L)	0.45 ± 0.25	0.56 ± 0.20	-0.102	0.052	0.050
Triglycerides (mmol/L)	0.99 ± 0.54	1.24 ± 0.43	-0.247	0.112	0.030
Atherogenic Index of plasma	-0.18 ±0.20	-0.05 ±0.19	-0.128	0.049	0.011
MDA (mmol/L)	0.74 ± 0.18	0.87 ± 0.23	-0.137	0.046	0.004
8-OHdg (ng/L)	9.2 ± 7.98	5.67 ± 1.36	3.550	1.256	0.006

Table 4 shows the correlation of duration of treatment and some other indices in leprosy patients undergoing multidrug therapy. Results showed a significant positive correlation between duration of treatment and TC (r = 0.611, p = 0.0001), HDL-C (r = 0.364, p = 0.048), LDL-C (r = 0.416, p = 0.022) and TG (r = 0.364, p = 0.048). However, a negative significant correlation was observed between duration of treatment and GSH (r = -0.401, p = 0.028).

**Table 4:** Correlation of duration of treatment and some other indices in leprosy patients undergoing multiple drug therapy.

Parameter	Index	r-value	p-value
Duration of treatment (months)	Body mass index	0.272	0.146
	Systolic BP	0.152	0.422
	Diastolic BP	-0.297	0.111
	Fasting plasma glucose	-0.077	0.687
	Total cholesterol	0.611	0.0001*
	HDL-cholesterol	0.364	0.048*
	LDL-cholesterol	0.416	0.022*
	VLDL-cholesterol	0.342	0.064
	Triglycerides	0.364	0.048*
	Atherogenic Index of plasma	0.166	0.379
	Total antioxidant capacity	0.230	0.221
	Total plasma peroxide	-0.331	0.074
	Oxidative Stress index	-0.260	0.165
	Malondialdehyde	-0.272	0.147
	Glutathione	-0.401	0.028*
	8-OHdg	0.148	0.148

*Significant at p<0.05

## Discussion

The study assessed fasting plasma glucose, lipid profile, total antioxidant capacity, total plasma peroxide, oxidative stress index, malondialdehyde, glutathione and 8-OHdg in patients with leprosy on mutltidrug therapy (MDT) and those relieved from therapy (RFT). The WHO health Organization has assiduously worked towards a leprosy-free world through its Global Leprosy Strategy 2016–2020 [33] and so has the National Tuberculosis and Leprosy Control Programme in Nigeria. However, more has to be done towards reintegrating those who have been cured of the disease into society. From our study, 60% of the leprosy patients that were relieved from treatment were unemployed. More than two thirds of the patients were males who should supposedly be breadwinners for their families.

Significantly lower levels of total cholesterol (TC), HDL-cholesterol (HDL-C) and LDL-cholesterol (LDL-C) were observed in patients on MDT and those RFT compared to the controls and significantly higher levels of VLDL-cholesterol (VLDL-C), triglycerides (TG) and Atherogenic index of plasma (AIP) in patients RFT compared to the controls. The low TC levels obtained in this study agrees with the findings of Sheikh *et al*., [34] and Nega *et al*., [35] who also detected lower TC levels in multibacillary form of the disease. However Silva *et al*., [16] reported normal levels of serum TC in both paucibacilliary and multibacilliary forms of the disease and other authors have even reported an increase in serum TC in patients with lepromatous leprosy [36]. The low serum TC observed in this study may be due to increase utilization of cholesterol by *M*. *leprae* for nutrition and persistence of the bacilli [18] or due to hepatic involvement as evidenced by several studies indicating hepatic damage by *M*. *leprae* bacilli [36]. It is also suggested that in lepromatous leprosy, free fatty acids are increased due to microbial modulation of lipoprotein lipase activity, which exerts an inhibitory effect on hepatic lipogenesis directly and indirectly on cholesterol synthesis by inhibiting pyruvate dehydrogenase enzymes of the liver [37].

The leprosy patients in both groups (MDT and RFT) had significantly lower HDL-C levels compared to the controls. This result differs from the findings of Silva *et al*., [16] and Sheikh *et al*. [34] who reported normal and significantly higher serum HDL-C levels in leprosy patients respectively but corroborate the findings of Gupta *et al*., [37] and Nega *et al*., [35] who detected significantly lower HDL-C levels in patients with the lepromatous form of the disease compared to the controls. It is suggested that this low serum HDL-C level observed could be due to increase in the levels of cytokines such as tumor necrosis factor (TNF-α) which lowers the HDL levels [35, 37]. Pro- inflammatory immune response against microbial infections up regulates the production of cytokines like TNF -α, IL-1, IL-2 & IL-6 as a host protective mechanism against bacterial assault [38]. TNF-α is a critical host-protective cytokine against mycobacterial diseases that decreases HDL levels [39] and studies have shown that TNF-α levels are increased in PB lesions and episodes of reverse reaction [40,41]. The precise mechanism by which TNF-α decrease HDL levels is uncertain and is likely to involve multiple mechanisms. It decreases the production of apo-A1, enhances HDL degradation by macrophages, decreases Lecithin Cholesterol Acyltransferase (LCAT) activity, causes an increase in serum amyloid A production, induces secretory phospholipase A2 (sPLA2) and endothelial cell lipase (HDL-metabolizing enzymes) which may all result in decreased HDL levels [38, 42].

Significantly lower levels of LDL-C were observed in multibacillary leprosy patients irrespective of drug therapy status when compared to the controls in this study. This agrees with the findings of a study Sheikh *et al*., [34]. Normal LDL-C levels were however observed by Silva *et al*. [16] in MB leprosy patients when compared to controls. In the present study, serum LDL-C level was also shown to be significantly lower in participants relieved from therapy when compared to those on MDT. The low LDL-C levels observed in this study may be due to impact of infection on hepatic lipogenesis. Nwosu & Nwosu [36] observed significantly high levels of alanine amino transferase in MB leprosy and Dhavalshankh *et al*., [43] observed significantly lower levels of albumin in lepromatous leprosy and these studies concluded that leprosy is associated with liver damage. The liver is the principal organ involved in the lipid metabolism and has a major role in the control of plasma LDL-C level since most LDL-C receptors are present in the liver; therefore its invasion by leprae bacilli may alter lipid metabolism [44].

This study showed significantly higher levels of VLDL and TG in MB leprosy patients relieved from treatment when compared to controls. This is similar to the work of Nega *et al*., [35]. However, normal triglyceride levels in leprosy patients with no significant variation in VLDL levels have been reported by other authors [16, 45]. The higher levels of triglyceride and VLDL-C cholesterol may be attributed to several mechanisms, including reduction of TG hydrolysis, lipopolysaccharide- and pro-inflammatory cytokine-induced de novo free fatty acid production and TG synthesis in the liver as well as reduction of lipoprotein lipase activity thus resulting in reduced VLDL clearance and increased TG levels [38]. The decreased clearance of TG-rich lipoproteins may be due to continued induction of cell-mediated inflammatory response caused by the presence of *M*. *leprae* antigens even after completion of therapy [46]. Some cytokines (TNF-α, IL-1, IL-2, IL-6) have been shown to decrease the synthesis of lipoprotein lipase, the key enzyme that metabolizes triglycerides in circulation, and inflammatory responses increases the production of these cytokines. Studies have also shown that inflammation also increases angiopoietin like protein 4, an inhibitor of lipoprotein lipase activity, which would further block the metabolism of VLDL and TG [47]. It may also be important to note that the increase in may also be due to age–related factors since those relieved from treatment were older than the controls, however this effect is mild as the statistical differences observed still remained after adjusting for age.

This work also showed positive significant correlation in TC, HDL-C and LDL-C levels with duration of treatment. This agrees with the observation that MDT may benefit hepatic lipogenesis and plasma lipid and lipoprotein abnormalities/levels may even return towards normal following recovery from the infection [42]. This study also showed significant increase in TG levels following increase in treatment duration. Some anti-inflammatory drugs have effects on lipid metabolism that are independent of the reduction in inflammation. For example, high dose glucocorticoid treatment results in an increase in serum triglyceride and VLDL levels due to the increased production and secretion of VLDL by the liver [42]. Because all MDT regimens for leprosy contain Clofazimine, which has strong anti-inflammatory activity [48], MDT may therefore increase VLDL secretion in the liver leading to increase in plasma TG levels.

Atherogenic index of plasma is a novel indicator of dyslipidemia and associated cardiovascular disease [49]. This study showed a significantly higher AIP in multibacillary leprosy patients when compared to controls. Inflammation and infections induce a variety of alterations in lipid metabolism that if prolonged could contribute to the increased risk of atherosclerosis. The most common changes are decreases in serum HDL-C and increases in triglyceride levels. In addition to affecting serum lipid levels, inflammation also adversely affects lipoprotein function. LDL is more easily oxidized as the ability of HDL to prevent the oxidation of LDL is diminished leading to atherosclerosis [42]. AIP was also significantly higher in the RFT group when compared to patients on MDT. This agrees with recent findings that cytokines mediated inflammatory responses, perhaps to persistent *M*. *leprae* antigen, still occur in leprosy patients even after being declared cured [35, 46]. Inflammation reduces serum HDL-C and increases TG levels and could contribute to the increased risk of atherosclerosis [42].

Results from this study also showed that the BMI of the both groups of leprosy patients compared to the controls was significantly lower indicating wasting, maybe due to malnutrition [12]. However, the WHR of leprosy patients relieved from treatment was significantly higher when compared to the control group. This agrees with the work of Silva *et al*., [16] who observed a higher waist circumference in multibacillary leprosy patients compared to controls. This suggests that WHR may be a better indicator of adiposity in these patients and leprosy.

The observed higher levels of TG, VLDL-C, AIP, WHR and lower levels of HDL-C observed in patients RFT compared to controls, suggests that leprosy patients who are relieved from treatment may be predisposed to developing a cardiovascular disease. This may result in a double burden of disease as well as increased mortality for those who suffer from this disease.

In this study oxidative stress markers assessed included total antioxidant capacity (TAC), total plasma peroxide (TPP), oxidative stress index (OSI), malondialdehyde (MDA), glutathione (GSH) while 8-hydroxy-2-deoxyguanosine (8-OHdg) was assessed as a marker of oxidative DNA marker. The significantly lower levels of TAC and GSH and higher levels of TPP, MDA in both groups of patients (MDT and RFT) and higher OSI and 8-OHdg in only the MDT group when compared to the controls suggests the presence of increased oxidative stress. These findings agree with the works of Prabhakar *et al*., [11] who showed that there is oxidative stress in MB leprosy cases, irrespective of their treatment status. Oxidative stress has been suggested to play a vital role in the pathogenesis of leprosy [12]. The leprosy patients exhibit increased oxidative stress which could mediate inflammatory episodes, depressed cell-mediated immune response, organ damage as well as degeneration of nerves in these patients [12]. Oxidative stress not only occurs in newly diagnosed cases but also in patients who have finished the therapy [50]. Higher MDA levels observed in RFT group suggests that there may be continued induction of ROS by persistent *M*. *leprae* antigen or remnant bacilli even after completion of therapy [46]. Significantly higher MDA levels indicate that increased lipid peroxidation and DNA damage due to free radical mediated injury occurs in leprosy patients [51]. A major defence against microbial infection is the macrophage system [52]. The macrophages infected by mycobacteria show increased phagocytosis, enzyme activity and oxygen consumption known as “respiratory burst,” [12]. Microbial killing by macrophages is associated with a burst of respiratory activity that also leads to production of ROS [51]. Another probable factor that may encourage lipid peroxidation in leprosy patients may the oxidative stress that is associated with amyloid formation and deposition [52]. Amyloid deposits have been reported in some leprosy patients with prevalence from autopsy studies ranging from 3.9% and 31% [53]. Major targets of ROS-associated peroxidation are the polyunsaturated fatty acids (PUFA) found in membrane lipids. PUFA is degraded by free radicals to form malondialdehyde (MDA) therefore MDA in serum serves as a marker of cellular damage due to free radicals [12]] and high serum MDA levels is indicative of increased lipid peroxidation and DNA damage by free radicals [54].

Antioxidant status is an index of oxidative stress. High level of oxidative stress is also shown by the low cellular antioxidant status [50] and decrease in antioxidant defense may be one of the reasons for increased levels of ROS and subsequent tissue damage in lepromatous spectrum [55]. In this study, both the RFT and MDT leprosy patients had lower TAC levels compared to controls although there was no significant variation in their GSH levels. Antioxidants which play a central role in maintaining the cells redox state both enzymatically and non-enzymatically by neutralizing free radicals, notably superoxide radicals, hydroxyl radicals, nitric oxide and carbon [56]. Studies have also shown that GSH is considered a potent inhibitor of lipid peroxidation process, and thus regulates the MDA content [57]. The comparable GSH levels in the leprosy patients compared with the controls contradicts findings in studies by Prabhakar *et al*., [11] and Osadalor and Okosun, [58] who all reported lower GSH levels in leprosy patients compared to controls. The reason for this is not clear.

Interestingly, there was significant negative correlation between GSH levels and duration of treatment. Decrease in GSH levels were observed with increase in treatment duration showing induction of oxidative stress with increased duration of MDT and only the MDT group had higher OSI and 8-OHdg compared to the controls. This may be due to the free radical producing effect of Dapsone, one of drugs in the MDT regimen [58]. Schlacher *et al*., [59] showed by their study that hydroxylation of Dapsone by hepatic cytochromes generate the metabolites (N-hydroxylamine DDS-NHOH and monoacetyl-hydroxylamine MADDS-NHOH) that produces free radicals by electron transfer. It may also have been utilized for replenishment of other crucial antioxidants such as vitamin E and C, which would get oxidized during the course of their antioxidant action [57]. They also suggested that MDT can thus reduce the activity of some antioxidant enzyme causing ROS accumulation and thereby exhausting the production of adaptive oxidants defenses, resulting in a higher oxidative stress index, lipid peroxidation as well as higher 8-OHdg levels in this group. The higher 8-OHdg suggests a predisposition of those undergoing treatment to oxidative DNA damage.

Our findings suggest that the increased oxidative stress observed may therefore be due to the body’s response to active infection; depletion of antioxidants defenses following therapy in the MDT and perhaps continued induction of free radicals by *M*. *leprae* antigens even after completion of therapy [46]. The increased OS observed in these patients can mediate inflammatory episodes, organ damage, depressed cell mediated immune response and degeneration of nerves in leprosy patients [12].

A limitation of this study was that we did not estimate the *M*. *leprae* bacterial load as this may have helped in shedding more light on the results obtained from those relieved from treatment i.e. to ascertain if there was persistence of infection. In addition, those relieved from treatment were significantly older than both the controls and those undergoing treatment. Though we tried to adjust for age, it may affect the interpretation of our result as age is a risk factor for cardiovascular disease and oxidative stress. Another limitation was the fact that we had no treatment–naïve group because this was not allowed by the hospital’s protocol because of the highly infectious nature of the disease. But it would have helped us observe the pattern of changes in the assessed analytes before, during and after treatment.

## Conclusion

This study shows that there are lower levels of total antioxidant capacity and higher levels of total plasma peroxide, malondialdehyde in leprosy patients undergoing multidrug therapy and those relieved from treatment and higher levels of 8-OHdg and oxidative stress index in leprosy patients undergoing multidrug therapy. This is indicative of increased oxidative stress, in multibacillary leprosy patients irrespective of drug treatment status and increased oxidative DNA damage in those undergoing treatment. Leprosy patients relieved from treatment may be susceptible to cardiovascular events as shown by higher levels of VLDL-cholesterol, triglycerides and atherogenic index of plasma observed in that group compared to controls. Antioxidants supplementation may be beneficial in the treatment of leprosy to protect against the effects of oxidative stress and clinical follow up on patients relieved from treatment may also be necessary to monitor health status and prevent development of cardiovascular events.

## Supporting information

S1 Data (XLSX)

## References

[1] World Health Organisation (2017). Guide lines for the diagnosis, treatment and prevention of leprosy. Executive summary. www.who.int. Retrieved 15/8/2019.

[2] Wangara F, Kipruto H, Ngesa O, Kayima J, Masini E, Sitienei J, Ngari F. The spatial epidemiology of leprosy in Kenya: A retrospective study. PLoS Negl Trop Dis. 2019;13(4):e0007329 10.1371/journal.pntd.0007329 31009481 PMC6497316

[3] The Leprosy Mission-Nigeria (2015). Facts and Questions about Leprosy. www.leprosymission-nig.org. Retrieved 15/8/2019.

[4] World health organisation (2017). Leprosy fact sheet, www.searo.who.int. Retrieved 1/8/2018

[5] World Health Organization. Global leprosy update, 2017: reducing the disease burden due to leprosy. Wkly Epidemiol Rec. 2018; 93 (35):445–456.

[6] World Health Organisation. Global leprosy update: accelerating reduction of disease burden. Wkly Epidemiol Rec. 2017;92(35):501–19. 28861986

[7] Muhammad F, Abdulkareem JH, Chowdhury AABM. South East Asia Journal of Public Health 2017;7(1):6–11.

[8] Nigeria Centre for Disease Control (2017). Leprosy. www.ncdc.gov.ng. Retrieved 13/9/2018.

[9] Fabel A, Giovanna Brunasso AM, Schettini AP, Cota C, Puntoni M, Nunzi E, Biondo G, Cerroni L, Massone C.Pathogenesis of Leprosy: An Insight Into BLymphocytes and Plasma Cells. Am J Dermatopathol. 2019; 41(6): 422–427. 10.1097/DAD.0000000000001310 30422829

[10] Schalcher TR, Borges RS, Coleman MD, Batista Júnior J, Salgado CG, Vieira JL, Romão PR, Oliveira FR, Monteiro MC. Clinical oxidative stress during leprosy multidrug therapy: impact of dapsone oxidation. PLoS One. 2014; 9(1):e85712 10.1371/journal.pone.0085712 24465659 PMC3899049

[11] Bassey IE, Ikpi DE, Isong IKP, et al Effect of combined calcium, magnesium, vitamin C and E supplementation on seminal parameters and serum oxidative stress markers in fructose-induced diabetic Wistar rats. Arch Physiol Biochem. 2020;1–8. 10.1080/13813455.2020.1716017 31983250

[12] Swathi M, Tagore R. Study of Oxidative Stress in Different Forms of Leprosy. Indian J Dermatol. 2015;60(3):321 10.4103/0019-5154.156426 26120177 PMC4458962

[13] Katerji M, Filippova M, Duerksen-Hughes P. Approaches and Methods to Measure Oxidative Stress in Clinical Samples: Research Applications in the Cancer Field. Oxid Med Cell Longev. 2019;2019:1279250 10.1155/2019/1279250 30992736 PMC6434272

[14] Moris D, Spartalis M, Spartalis E, Karachaliou GS, Karaolanis GI, Tsourouflis G, Tsilimigras DI, Tzatzaki E, Theocharis S. The role of reactive oxygen species in the pathophysiology of cardiovascular diseases and the clinical significance of myocardial redox. Ann Transl Med. 2017; 5(16):326 10.21037/atm.2017.06.27 28861423 PMC5566734

[15] Rafieian-Kopaei M, Setorki M, Doudi M, Baradaran A, Nasri H. Atherosclerosis: process, indicators, risk factors and new hopes. Int J Prev Med. 2014;5(8):927–46. 25489440 PMC4258672

[16] Silva RVG, de Araújo RS, Aarão TLS, da Silva Costa PD, Sousa JR, Quaresma JAS. Correlation between therapy and lipid profile of leprosy patients: is there a higher risk for developing cardiovascular diseases after treatment?. Infect Dis Poverty. 2017;6(1):82 10.1186/s40249-017-0295-1 28457229 PMC5410692

[17] De Rosa S, Arcidiacono B, Chiefari E, Brunetti A, Indolfi C, Foti DP. Type 2 Diabetes Mellitus and Cardiovascular Disease: Genetic and Epigenetic Links. Front Endocrinol (Lausanne). 2018 1 17;9:2 10.3389/fendo.2018.00002 29387042 PMC5776102

[18] Marques MA, Berrêdo-Pinho M, Rosa TL, Pujari V, Lemes RM, Lery LM, Silva CA, Guimarães AC, Atella GC, Wheat WH, Brennan PJ, Crick DC, Belisle JT, Pessolani MC. The Essential Role of Cholesterol Metabolism in the Intracellular Survival of Mycobacterium leprae Is Not Coupled to Central Carbon Metabolism and Energy Production. J Bacteriol. 2015 12;197(23):3698–707. 10.1128/JB.00625-15 26391209 PMC4626898

[19] World Medical Association: Declaration of Helsinki. http://www.wma.net/en/30publications/10policies/b3/17c.pdf

[20] Chew GT, Gan SK, Watts GF. Revisiting the metabolic syndrome. Med J Aust 2006; 185 (8): 445–449. 17137436 10.5694/j.1326-5377.2006.tb00644.x

[21] Trinder P. Determination of blood glucose using an oxidase-peroxidase system with a non-carcinogenic chromogen. J Clin Pathol. 1969; 22(2):158–161. 10.1136/jcp.22.2.158 5776547 PMC474026

[22] Allain CC, Poon LS, Chan CS, Richmond W, Fu PC. Enzymatic Determination of Total Serum Cholesterol. Clin Chem. 1974; 20(4):470–475. 4818200

[23] Bucolo G. David H. Quantitative Determination of Serum Triglycerides by the Use of Enzymes. Clin Chem. 1973; 19(5):476–482. 4703655

[24] Demacker PN, Hessels M, Toenhake-Dijkstra H, Baadenhuijsen H. Precipitation methods for high-density lipoprotein cholesterol measurement compared, and final evaluation under routine operating conditions of a method with a low sample-to-reagent ratio. Clin Chem. 1997; 43(4):663–668. 9105270

[25] Friedewald WT, Levy RI, Fredrickson DS. Estimation of the Concentration of Low-Density Lipoprotein Cholesterol in Plasma, Without Use of the Preparative Ultracentrifuge. Clin Chem. 1972;18(6):499–502 4337382

[26] Burtis C. A., Ashwood E. R. & Bruns D. E. (2008). *Tietz Fundamentals of Clinical chemistry* (6th Eds.). Elsevier: New York Pp 125.

[27] Dobiasova M, Frohlich J, Sedova M, Cheung MC, Brow BG. Cholesterol esterification and atherogenic index of plasma correlate with lipoprotein size and findings on coronary angiography. *J*. *Lipid Res*, 2011; 52(3): 566–571. 10.1194/jlr.P011668 21224290 PMC3035693

[28] Ellman GL. Tissue sulfhydryl groups. Arch Biochem Biophys. 1959; 82(1):70–77. 10.1016/0003-9861(59)90090-6 13650640

[29] Buege JA, Aust SD. Microsomal lipid peroxidation. Methods Enzymol. 1978;52:302–310. 10.1016/s0076-6879(78)52032-6 672633

[30] Koracevic D, Koracevic G, Djordjevic V, Andrejevic S, Cosic V. Method for the measurement of antioxidant activity in human fluids. J Clin Pathol. 2001;54(5):356–61. 10.1136/jcp.54.5.356 11328833 PMC1731414

[31] Miyazawa T. Determination of phospholipid hydroperoxides in human blood plasma by a chemiluminiscence-HPLC assay. Free Radic Biol Med. 1989;7(2):209–217. 10.1016/0891-5849(89)90017-8 2806945

[32] Harma M., Harma M, Erel O. (2003). Increased Oxidative Stress in patients with hydratidiform mole. Swiss Med Wkly. 2003;133(41–42):563–566. 14691728 10.4414/smw.2003.10397

[33] Blok DJ, De Vlas SJ, Richardus JH. Global elimination of leprosy by 2020: are we on track?. Parasit Vectors. 2015;8:548 Published 2015 Oct 22. 10.1186/s13071-015-1143-4 26490878 PMC4618543

[34] Sheikh GS, Zubari NA., Sheikh MH, Abro MR. Evaluation of lipid profile in leprosy patients. Medical Forum Monthly, 2012; 23 (12):48–50

[35] Nega, T. (2016). "Immunological and lipid profile among leprosy patients at ALERT Centre, Addis Ababa Ethiopia". A thesis submitted to the school of graduate studies of Addis Ababa University in partial fulfilment of the requirements for the Degree of Masters of science in clinical laboratory science, Diagnostic and Public Microbiology special track.Mmmm

[36] Nwosu CM, Nwosu SNN. Abnormalities in Serum Lipids and Liver Fuction in Nigeria Patients with Leprosy. Journal of Medical Investigation and Practice. 2001; 2:5–10

[37] Gupta A, Koranne RV, Kaul N. Study of serum lipids in leprosy. Indian J Dermatol Venereol Leprol 2002; 68:262–266. 17656962

[38] Yu JE, Han SY, Wolfson B, Zhou Q. The role of endothelial lipase in lipid metabolism, inflammation, and cancer. Histol Histopathol. 2018 1;33(1):1–10. 10.14670/HH-11-905 28540715 PMC5858721

[39] Inoue M., Niki M., Ozeki Y, Nagi S, Chadeka EA, Yamaguchi T, Osada-Oka M, Ono K, Oda T, Mwende F, Kaneko Y, Matsumoto M, Kaneko S, Ichinose Y, Njenga SM., Hamano S, Matsumoto S. High-density lipoprotein suppresses tumor necrosis factor alpha production by mycobacteria-infected human macrophages. Sci Rep 2018; 8: 6736 10.1038/s41598-018-24233-1 29712918 PMC5928146

[40] Yuniati R, Munita F F, Dayana B, Amalia F. Difference of Tumor Necrosis Factor (TNF-α) Levels in Multibacillary Leprosy between Reversal Reaction and Non-reversal Reaction Patients. Pak J Med Health Sci. 2018; 12(3): 1381–1383.

[41] Madan NK, Agarwal K, Chander R. Serum cytokine profile in leprosy and its correlation with clinico-histopathological profile. Lepr Rev. 2011;82(4):371–382. 22439277

[42] Feingold KR, Grunfeld C. The Effect of Inflammation and Infection on Lipids and Lipoproteins [Updated 2019 1 8]. In: Feingold KR, Anawalt B, Boyce A, et al, editors. Endotext [Internet]. South Dartmouth (MA): MDText.com, Inc.; 2000-. https://www.ncbi.nlm.nih.gov/books/NBK326741/

[43] Dhavalshankh GP, Dhavalshankh AG, Gaurkar S. Assessment and Comparison of Liver Functions in Leprosy. Int J Cur Res Rev. 2017; 9(5):26–32.

[44] Mattos KA, Oliveira VC, Berrêdo-Pinho M, Amaral JJ, Antunes LC, Melo RC, Acosta CC, Moura DF, Olmo R, Han J, Rosa PS, Almeida PE, Finlay BB, Borchers CH, Sarno EN, Bozza PT, Atella GC, Pessolani MC. Mycobacterium leprae intracellular survival relies on cholesterol accumulation in infected macrophages: a potential target for new drugs for leprosy treatment. Cell Microbiol. 2014;16(6):797–815. 10.1111/cmi.12279 24552180 PMC4262048

[45] Eichelmann K, González González SE, Salas-Alanis JC, Ocampo-Candiani J. Leprosy: Update, Definition, Pathogenesis, Classification, Diagnosis and Treatment. Actas Dermosifiliogr. 2013;104(7):554–63. 10.1016/j.adengl.2012.03.028 23870850

[46] Lambert S., Walker S. & Harnisch J. (2017). Leprosy (Hansen’s Disease) *The Travel and Tropical Medicine Manual* (5th eds.). Elsevier Inc: New York Pp 513–523

[47] La Paglia L, Listì A, Caruso S, Amodeo V, Passiglia F, Bazan V, Fanale D. Potential Role of ANGPTL4 in the Cross Talk between Metabolism and Cancer through PPAR Signaling Pathway. PPAR Res. 2017;2017:8187235 10.1155/2017/8187235 28182091 PMC5274667

[48] Cruz RCDS, Bührer-Sékula S, Penna MLF, Penna GO, Talhari S. Leprosy: current situation, clinical and laboratory aspects, treatment history and perspective of the uniform multidrug therapy for all patients. An Bras Dermatol. 2017; 92(6):761–773. 10.1590/abd1806-4841.20176724 29364430 PMC5786388

[49] Zhu X, Yu L, Zhou H, Ma Q, Zhou X, Lei T, Hu J, Xu W, Yi N, Lei S. Atherogenic index of plasma is a novel and better biomarker associated with obesity: a population-based cross-sectional study in China. Lipids Health Dis. 2018;17(1):37 10.1186/s12944-018-0686-8 29506577 PMC5836428

[50] Vijayaraghavan R, Paneerselvam C. Erythrocyte Antioxidant Enzymes in Multibacillary Leprosy Patients. International Journal of Applied Biology and Pharmaceutical Technology. 2011; 2 (2): 409–413.

[51] Garad AS, Suryakar AN, Shinde CB. Oxidative Stress And Role Of Thiol In Leprosy. International Journal of Pharmaceutical, Biological and Chemical Sciences. 2014; 3 (2): 22–26

[52] Zraika S, Hull RL, Udayasankar J, et al Oxidative stress is induced by islet amyloid formation and time-dependently mediates amyloid-induced beta cell apoptosis. Diabetologia. 2009;52(4):626–635. 10.1007/s00125-008-1255-x 19148619 PMC2719780

[53] Sanz-Martín N, Samillán-Sosa Kdel R, De Miguel J, Martínez-Miguel P. Renal amyloidosis in leprosy, an infrequent cause of nephrotic syndrome in Europe. *BMJ Case Rep*. 2016;2016:bcr2016216038. Published 2016 Aug 3. 10.1136/bcr-2016-216038 27489069 PMC4986063

[54] Trimbake SB, Sontakke AN, Dhat VV. Oxidative stress and antioxidant vitamins in leprosy. Int J Res Med Sci. 2013; 1(3): 100–104

[55] Jyothi P, Riyaz N, Nandakumar G, Binitha M P. A study of oxidative stress in paucibacillary and multibacillary leprosy. Indian J Dermatol Venereol Leprol 2008;74:80 10.4103/0378-6323.38428 18188882

[56] Kurutas EB. The importance of antioxidants which play the role in cellular response against oxidative/nitrosative stress: current state. Nutr J. 2016;15(1):71 10.1186/s12937-016-0186-5 27456681 PMC4960740

[57] Chhabra N, Bhattacharya SN, Singal A, Ahmed RS, Verma P. Profile of oxidative stress in response to treatment for Type 1 leprosy reaction. Lepr Rev. 2015; 86(1):80–88. 26065150

[58] Osadolor HB, Okosun R. Non-enzymatic antioxidants status of leprosy patients in a leprosarium settlement in Nigeria. Biokemistri. 2014; 26(2): 50–54.

[59] Schalcher TR, Vieira JL, Salgado CG, Borges Rdos S, Monteiro MC. Antioxidant factors, nitric oxide levels, and cellular damage in leprosy patients. Rev Soc Bras Med Trop. 2013 Sep-Oct;46(5):645–9. 10.1590/0037-8682-1506-2013 24270257

