# Supplementary material for: Cardiovascular disease risk factors and markers of oxidative stress and DNA damage in leprosy patients in Southern Nigeria
Source: PLoS Negl Trop Dis. 2020 Oct 12;14(10):e0008749. doi: 10.1371/journal.pntd.0008749 (PMC7580906; doi:10.1371/journal.pntd.0008749)
Supplement: Attachment — Submitted filename: PLOS Neglected Tropical Diseases response to reviewers.docx [file pntd.0008749.s002.docx]

I thank you for the extensive review and points raised and I have taken care of each point as follows

Reviewer #1: To strengthen the work, it is necessary to align the analyzes carried out with a well-defined hypothesis and objective. I suggest that the authors include the purpose of the study in the introduction.

Answer: Thank you. This has been inserted in lines 81 to 85

In lines 102-105 the authors specify details about the collection of information and anthropometric data, however, they do not mention the measurement of blood pressure and the calculation of the waist-to-hip ratio. I suggest including this information as well as the reference values associated with it.

Thank you. This has been inserted in lines 113 to 121

The absence of reference values for the evaluated markers made it difficult to read and analyze the tables, so I suggest the authors to detail this information.

Answer: reference values for the evaluated markers have been added

Exclusion criteria topics can be added to the inclusion criteria topic. Lines 108-112 repeat some of the information mentioned in the previous topic (Study design and subject selection). I suggest that the authors review the need for repetition of information.

Answer: The topics have been merged together and repeated information deleted

In the statistical analysis, the authors do not inform whether they performed the assessment of the normality of the data and which test was used. This information must be clear since it supports the choice for parametric tests. I also suggest that the authors specify which regression analysis was used.

The assessment of the normality of the data was done using the Shapiro-Wilk’s test. Regression analysis was not done. That was a typographical error.

Reviewer #2: The objectives and hypothesis are clearly stated and the study design is appropriate to address the objective. Overall, the population is clearly described. No sample size calculation was given, but there seems to be a sufficient number of participants in each group (n = 30). The statistical analysis support the conclusions, however it is not clear whether the groups have similar characteristics (e.g. age, sex distribution). Ethical permission has been received and this was reported.

The groups have similar sex distribution, but those relieved from treatment are older than both the controls and those undergoing treatment.

Reviewer #3: Well defined and nicely described methodology section of the study

Thank you

--------------------

<b>Results</b></br></br>

-Does the analysis presented match the analysis plan?</br>

-Are the results clearly and completely presented?</br>

-Are the figures (Tables, Images) of sufficient quality for clarity?</br></br>

Reviewer #1: The results follow the analysis plan. I recommend the authors briefly explore the differences between groups (MDT, RFT, and controls) for the results presented in Tables 1 and 2.

Answer: We have carried out a chi squared test of independence which has enabled us to explore some of the variables in Table 1. This is shown in the results section.

Table 1, which presents the sociodemographic data of the study subjects, did not include information on the age variable. The inclusion of this variable is relevant, as it is an important factor in the analysis of cardiovascular risk and oxidative stress.

Answer: the age of the subjects have been included

The analysis of the waist-to-hip ratio without stratifying according to gender makes difficult to interpret since it has different reference values between men and women.

Answer: Yes this is true, but we thought since the sex distribution was similar among groups it would not affect it. We actually went further to see the effect of gender on the parameters and there was none.

Reviewer #2: The analysis match the analysis plan. The results are presented, but there is some information missing in table 1 (e.g. compliance) and the scale/value of some variables is not clear (e.g. duration of treatment in days/weeks/months?). The quality of the tables is sufficiently.

Answer: Thank you. Compliance, duration of treatment (months) and duration relieved from treatment (years) has been added to Table 1

Reviewer #3: Nicely described results section as well as nicely presented analysis of results.

Thank you

--------------------

<b>Conclusions</b></br></br>

-Are the conclusions supported by the data presented?</br>

-Are the limitations of analysis clearly described?</br>

-Do the authors discuss how these data can be helpful to advance our understanding of the topic under study?</br>

-Is public health relevance addressed?</br></br>

Reviewer #1: The discussion presented is interesting and states the limitations and generalizations of the study.

In lines 263-269, the authors discuss the role of pro-inflammatory cytokines in decreasing HDL levels. Since the patients included had multibacillary leprosy, the literature refers that the immune response elicited produces predominantly cytokines from the Th2 pattern. Studies have shown that TNF-α levels are increased in PB lesions and episodes of reverse reaction. These information should be taken into consideration when elaborating on the topic.

Answer: Thank you. That has been included with relevant references

Reviewer #2: The conclusions are supported by the data presented and are clearly stated. The limitations of the analysis are clearly described and the authors show a great understanding of the topic. The public health relevance is addressed and recommendations are made for interventions.

Answer: Thank you

Reviewer #3: Conclude the study section very good and given a key message for leprosy control program.

Answer: Thank you

--------------------

<b>Editorial and Data Presentation Modifications?</b></br><br/>

Use  this section for editorial suggestions as well as relatively minor modifications of existing data that would enhance clarity. If the only modifications needed are minor and/or editorial, you may wish to recommend “Minor Revision” or “Accept”.

Reviewer #1: Abstract

Remove the abbreviation ROS, as the word is mentioned only once in the summary;

Answer: It has been removed

The abbreviations BMI, TC, WHR, and AIP have no mention of the original word.

The original words have been included.

Introduction

Line 45-46: specify the period to which the total number of people infected with leprosy refers to the sentence;

Answer: the period has been included

Line 52: present the terms referred to in the abbreviation ROS.

Answer: this has been done

Methodology

Line 126: add the abbreviation FPG to the term fasting plasma glucose.

Answer: It has been added

Results

Line 182: mean BPD values were significantly lower in the MDT group when compared to the control group;

Answer: Thank you. It has been corrected

Line 185: correct the p-value for the TAC.

Answer: Thank you. It has been corrected

Reviewer #2: Minor revisions are needed for the methods, results and discussion.

I recommend to make the following revisions:

Methods:

Please describe what materials and methods you used. For example in line 118 'whole blood was collected' how did you do this and with what type of material from what company?

Also for methods that are described elsewhere like line 126 'method of Trinder' did you use the exact same material from the exact same company and distribution center?

Answer: Thank you. That is the method on which the commercial kit we obtained was based i.e. the glucose oxidase method. The Randox kits were used.

What do you mean with minor modifications in line 141?

Answer: With minor modifications as described by Harma et al, 2003

Please provid details on your protocols. For example in line 122 'frozen until assayed' at what temperature, -80 or -20?

Answer: It was stored at -20^0^C

Specify the ELISA kit you used and from what company, to do this correctly you need to give the companies full name, the city/state the company is located and the country the company is located and put this within brackets behind the material. So for example: Agisera's ELISA kit no:12345 (Agisera, Vännäs, Sweden).

This also applies to the software you use SPSS in line 155 and Microsoft in line 160

Results

Table 1: Did you test for differences between the groups, for example in gender distribution? And what about the age of the patients? It seems like there might be a difference in the gender distribution, did you check whether this affected your results and did you correct for this accordingly in your results?

Answer Yes I did there was no significant distribution in gender distribution, however the leprosy patients relieved from treatment were older than those undergoing treatment and controls

Table 1: Not necessary to put disease form and HIV in it, since you selected for MB patients and for no other ilnesses, right?

Answer: Yes, thank you. It has been removed

Table 1: I miss the compliance in the table, this is described in line 166.

Answer: Yes, thank you. It has been included in Table 1

Table 1: how did you assess physical deformities, on what scale? This is also not described in the methods.

We just noted if they were any deformities present but we did not scale them since we did not look at severity of disease.

Table 2: Are the Calf F and Crit. F really needed?

Answer: It has been removed

Table 4: The duration of treatment is in days/weeks/moths/years?

Answer: In months, this has been included in Tables 1 and 4

Discussion:

Line 250 : 'both forms' what forms is meant by this. Be careful with the different 'forms' of leprosy, leprametous and MB are different terms since they belong to different classification systems but these terms overlap.

Answer: it has been clarified and now reads “both paucibacilliary and multibacilliary forms of the disease”

Line 365 - 368: What might be causing these different results?

Answer: the reason is not clear. Actually when we pooled all the leprosy patients together regardless of treatment status, GSH levels were significantly lower than those of controls (p = 0.036). However when we divided them into different groups and used ANOVA it was not significant even after doing a post hoc . please take a look at a sample of the Table (vot included in manuscript

**Age, anthropometric indices, blood pressures, lipid profile, atherogenic index of plasma and oxidative stress markers in leprosy patients and controls**

| **Parameter** | **Leprosy Patients n=60** | **Controls n=30** | **Calc t** | **Crit. t** | **p-value** |
| --- | --- | --- | --- | --- | --- |
| Age (yr) | 44.4 ± 15.20 | 38.1± 13.02 | 1.925 | 1.98 | 0.057 |
| BMI (kg/m^2^) | 20.0 ± 4.10 | 23.6± 2.58 | -4.356 | 1.98 | 0.0001* |
| Waist/Hip ratio | 0.87 ±0.06 | 0.85 ±0.05 | 2.048 | 1.98 | 0.044* |
| Systolic BP (mmHg) | 118.0 ±19.36 | 116.8 ±6.58 | 0.320 | 1.98 | 0.750 |
| Diastolic BP (mmHg) | 72.9 ±10.10 | 78.2 ±9.74 | 2.367 | 1.98 | 0.020* |
| FPG (mmol/L) | 5.29 ± 5.19 | 4.42 ± 0.47 | 0.905 | 1.98 | 0.368 |
| Total cholesterol (mmol/L) | 3.69 ± 0.94 | 4.68 ± 0.71 | 5.079 | 1.98 | 0.0001* |
| HDL-cholesterol (mmol/L) | 1.39 ± 0.37 | 1.65 ± 0.41 | 3.016 | 1.98 | 0.003* |
| LDL-cholesterol (mmol/L) | 1.82 ± 0.84 | 2.61 ± 0.58 | 4.620 | 1.98 | 0.0001* |
| VLDL-cholesterol (mmol/L) | 0.51 ± 0.23 | 0.45 ± 0.14 | 1.304 | 1.98 | 0.196 |
| Triglycerides (mmol/L) | 1.12 ± 0.50 | 0.98 ± 0.30 | 1.378 | 1.98 | 0.172 |
| Atherogenic Index of plasma | -0.11 ±0.21 | -0.23 ±0.18 | 2.667 | 1.98 | 0.009* |
| MDA (mmol/L) | 0.81 ± 0.22 | 0.52 ± 0.10 | 6.736 | 1.98 | 0.0001* |
| GSH (mg/dL) | 79.2 ± 20.32 | 90.6 ± 29.70 | 2.136 | 1.98 | 0.036* |

Results expressed as Mean ± SD

*significant at p<0.05

Reviewer #3: Not required

--------------------

<b>Summary and General Comments</b></br></br>

Use  this section to provide overall comments, discuss strengths/weaknesses of the study, novelty, significance, general execution and scholarship. You may also include additional comments for the author, including concerns about dual publication, research ethics, or publication ethics. If requesting major revision, please articulate the new experiments that are needed.

Reviewer #1: It is a relevant study that contributes to the improvement of care for patients with leprosy by bringing evidence about the increase in cardiovascular risk and oxidative stress in MB patients in Nigeria. It adds knowledge about the theme and points to the need to monitor cardiovascular comorbidities in patients on MDT and released from therapy.

Reviewer #2: Overall, a very interesting manuscript! The introduction and discussion are well-written and show great understanding of the topic. This is very valuable since it is the first study in Africa evaluating oxidative status in leprosy patients, while leprosy is still prevalent in parts of this continent. The results are very interesting and relevant and the study has been performed according to ethical guidelines.

Reviewer #3: The manuscript Number PNTD-D-20-00597, entitled "Cardiovascular Risk Factors and Markers of Oxidative Stress and DNA Damage in Leprosy Patients in Southern Nigeria”. This is an interesting and exhaustive article on the cardiovascular risk factors in leprosy patients. The article is relatively well written. However, there are various fallacies in this article.

I would like the authors to consider the following points to make before this article can be processed further.

Comments on the manuscript PLOSNTD- major revision

01. How was the Multibacillary (MB) leprosy cases diagnosed?

Answer: the Physicians at the leprosy center diagnosed these cases after routine checks and diagnosis. So we actually made use of known leprosy cases and got relevant information from their case files.

02. The treatment for MB leprosy cases should be described in detail, including dosage and duration.

Answer: that has been done

03. How was the cardiac evaluation done?:

Cardiac evaluation was not done. What we did was a measurement of cardiovascular disease risk factors, including anthropometric indices, blood pressure, lipid profile and atherogenic index of plasma. The topic has been modified to reflect that.

04. Was ECG and Echocardiography as well as X-ray chest done for evaluation of cardiac status?

Answer: No. This was not in the scope of our study

05. What could have been the exact cause of oxidative stress and DNA damage?

Answer: Oxidative metabolic activity associated with “respiratory burst” by macrophages while combating mycobacterial infection; chronic inflammation as well as the free radical producing effect of drugs in the MDT regimen especially Dapsone.

06. Amyloidosis is an important cause of cardiac, renal and hepatic deterioration in MB leprosy patients. So mention has to be made of this entity in the discussion section.

Answer Mention has been made of amyloidosis and its role in contributing to oxidative stress in leprosy patients.

07. In the introduction section, Epidemiology of the disease should be briefly described, including the number of cases worldwide as well as in Nigeria.

Answer: this was done in the introduction

08. Mention has to be made about the leprosy elimination program in the world as well as in Africa and Nigeria in the discussion section.

I have made mention of the leprosy elimination program in the world as well as Nigeria.

09. The English and grammatical errors need to be corrected.

Answers: These have been corrected

All the above mentioned points need to be taken care of before further processing.

Answer: All done

--------------------
