# Supplementary material for: Cardiovascular disease risk factors and markers of oxidative stress and DNA damage in leprosy patients in Southern Nigeria
Source: PLoS Negl Trop Dis. 2020 Oct 12;14(10):e0008749. doi: 10.1371/journal.pntd.0008749 (PMC7580906; doi:10.1371/journal.pntd.0008749)
Supplement: Attachment — Submitted filename: PLOS Neglected Tropical Diseases response to reviewers 2.docx [file pntd.0008749.s003.docx]

Response to reviewers

Key Review Criteria Required for Acceptance?

As you describe the new analyses required for acceptance, please consider the following:

Methods-Are the objectives of the study clearly articulated with a clear testable hypothesis stated?

-Is the study design appropriate to address the stated objectives?

-Is the population clearly described and appropriate for the hypothesis being tested?

-Is the sample size sufficient to ensure adequate power to address the hypothesis being tested?

-Were correct statistical analysis used to support conclusions?

-Are there concerns about ethical or regulatory requirements being met?

Reviewer #1: Yes.

Reviewer #2: The methods are much more clear now that details have been added on the procedures and materials.

Reviewer #3: Described methodology of the study is well defined.

Answer: Thank you

--------------------

Results

-Does the analysis presented match the analysis plan?

-Are the results clearly and completely presented?

-Are the figures (Tables, Images) of sufficient quality for clarity?

Reviewer #1: Yes.

Reviewer #2: As expected, the socio-demographic characteristics of the groups significantly differ. These characteristics can have major impact on the outcome parameters measured in this study. The increased aged in the group that is relieved from therapy can for example affect the results significantly. If I understand it correctly, these characteristics have not been taking along in the analysis of the outcome parameters, while they should be in my opinion.

Answer: that has been done

In addition, the paragraph describing table 2 and 3 (line 207-226) is really hard to read because of all the p-values in the text.

Answer: Yes it’s true, but unfortunately the p values for most of the variables are different so I can’t group them together and I need to state them

Finally the last parameter in table 2 'duration relieved from therapy' is probably displaced, because now it is placed under the group that still receives therapy.

Answer: Thank you. That has been taken to the group that has been relieved from therapy. Please see line 253

Reviewer #3: Results section is nicely presented.

Answer: Thank you

--------------------

Conclusions

-Are the conclusions supported by the data presented?

-Are the limitations of analysis clearly described?

-Do the authors discuss how these data can be helpful to advance our understanding of the topic under study?

-Is public health relevance addressed?

Reviewer #1: Yes.

Answer: Thank you

Reviewer #2: Only few limitations have been described, while this study has multiple. Especially the fact that the characteristics of the groups significantly differ and that this might affect the results is important to mention in the discussion. Please elaborate the limitations in the discussion.

Answer: I have added that as a limitation in lines 447 - 450

Reviewer #3: The conclusion section of the study is excellent and forward a key message for leprosy control program.

Answer: Thank you

--------------------

Editorial and Data Presentation Modifications?<br/>

Use this section for editorial suggestions as well as relatively minor modifications of existing data that would enhance clarity. If the only modifications needed are minor and/or editorial, you may wish to recommend “Minor Revision” or “Accept”.

Reviewer #1: The manuscript needs formatting, in some sentences, there is an excess of periods (lines 201, 300), excessive use of spaces (lines 175,133, 136, etc.) and a different color in the text (269-273).

Answer: Thank you. Excess of periods in lines 179, 201, 300 and 530 have been removed

Excessive use of spaces in the whole document have been corrected

Reviewer #2: Please correct for the differences between the groups a priori (e.g. age, deformities) during your analysis of the outcome parameters. There is clearly a link between several cardiovasculair risk factors/markers of oxidative stress and age. So if the groups differ so significantly (p = 0.001) you can not 'just' compare the outcome parameters of these groups as you did in table 3. Please also discuss this in the discussion within the limitation section.

Answer: Thank you. We have done that by adding age as a covariate into the analysis and the results did not change (we used the ANCOVA model using GLM procedure). It has been indicated in line 214 to 216. It has also been incorporated in the discussion (please see line 350 – 353). I have also discussed this within the limitation section (please see line 447 – 450).

Reviewer #3: Not required

--------------------

Summary and General Comments

Use this section to provide overall comments, discuss strengths/weaknesses of the study, novelty, significance, general execution and scholarship. You may also include additional comments for the author, including concerns about dual publication, research ethics, or publication ethics. If requesting major revision, please articulate the new experiments that are needed.

Reviewer #1: The authors did a great job of reviewing and endeavored to improve the manuscript. The reviewers' suggestions were taken into account, and the doubts raised were clarified.

Answer: Thank you

Reviewer #2: No comments

Answer: Thank you

Reviewer #3: The manuscript Number PNTD-D-20-00597, entitled "Cardiovascular Risk Factors and Markers of Oxidative Stress and DNA Damage in Leprosy Patients in Southern Nigeria”. The revised article is relatively well written.

Answer: Thank you

--------------------
